# Supplementary figures and images for: A harmonized single-cell RNA-seq atlas of human localized and metastatic prostate cancers and benign tissues
Source: bioRxiv. 2026 May 20:2026.05.18.725966. Preprint. [Version 1] doi: 10.64898/2026.05.18.725966 (PMC13228276; doi:10.64898/2026.05.18.725966)

# Cho et al. Supp Figure 1

**a**

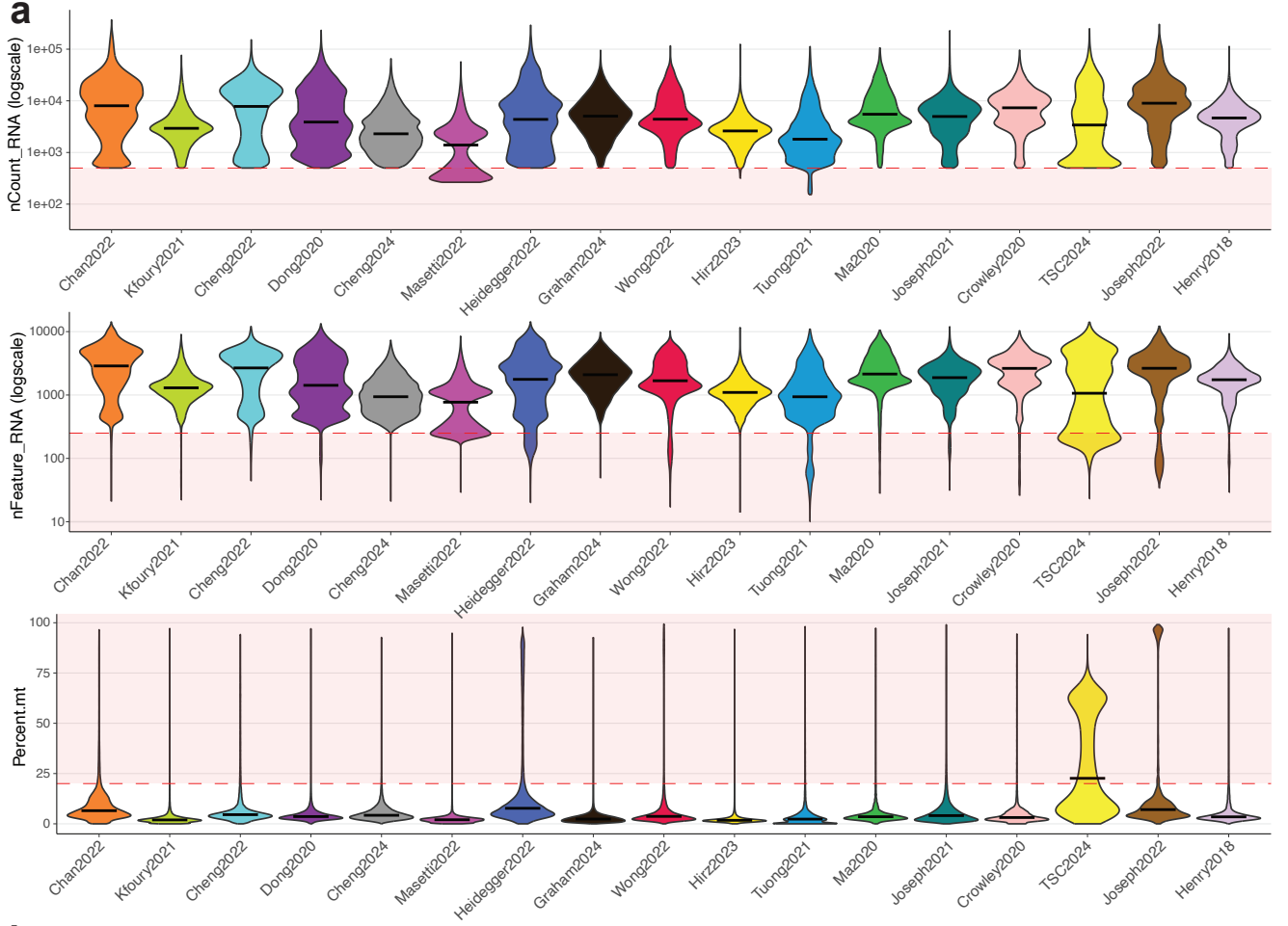

**b**

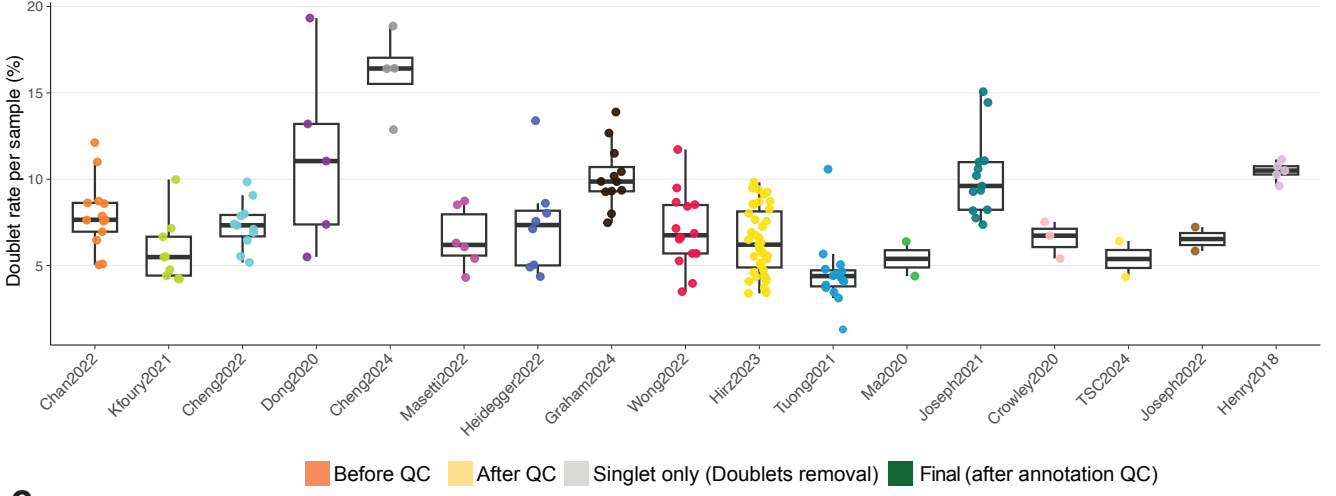

**c**

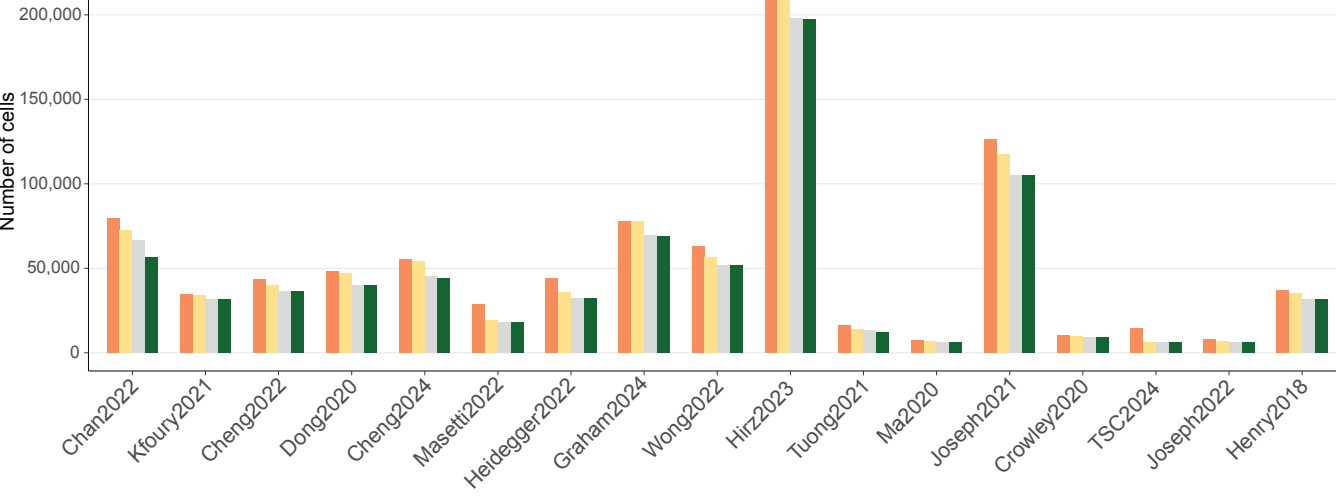

Cho et al. Suppl Figure 2

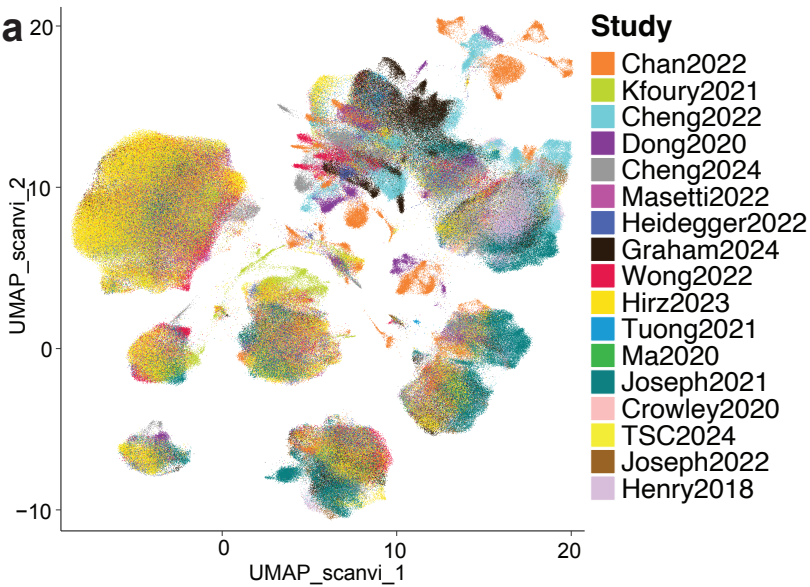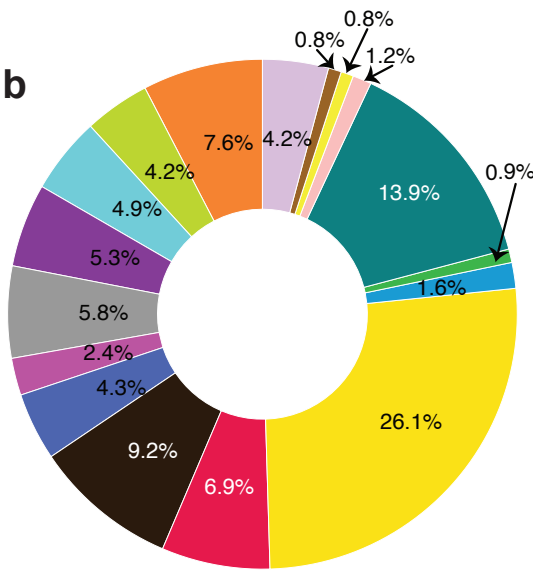

Cho et al. Suppl Figure 3

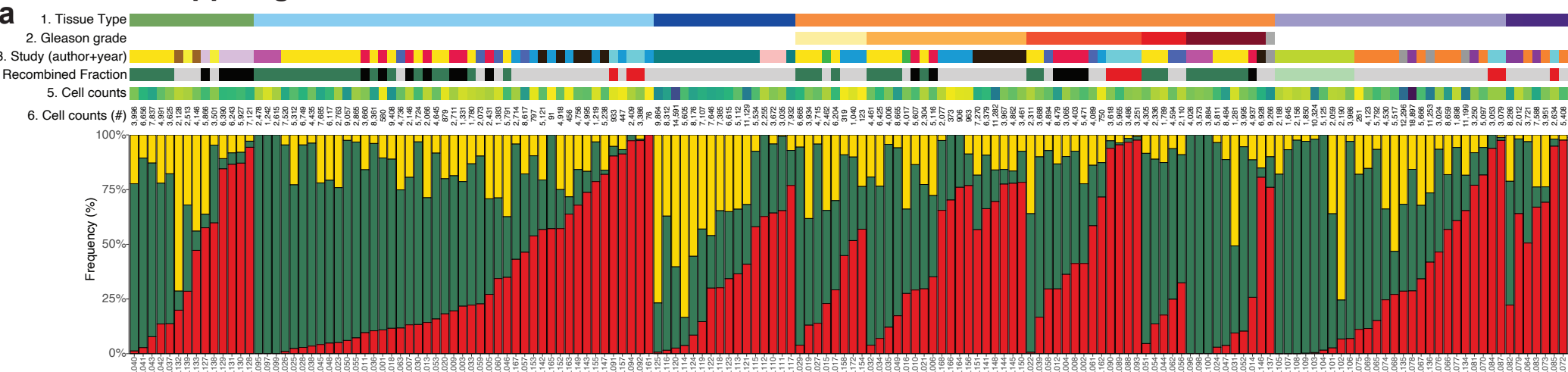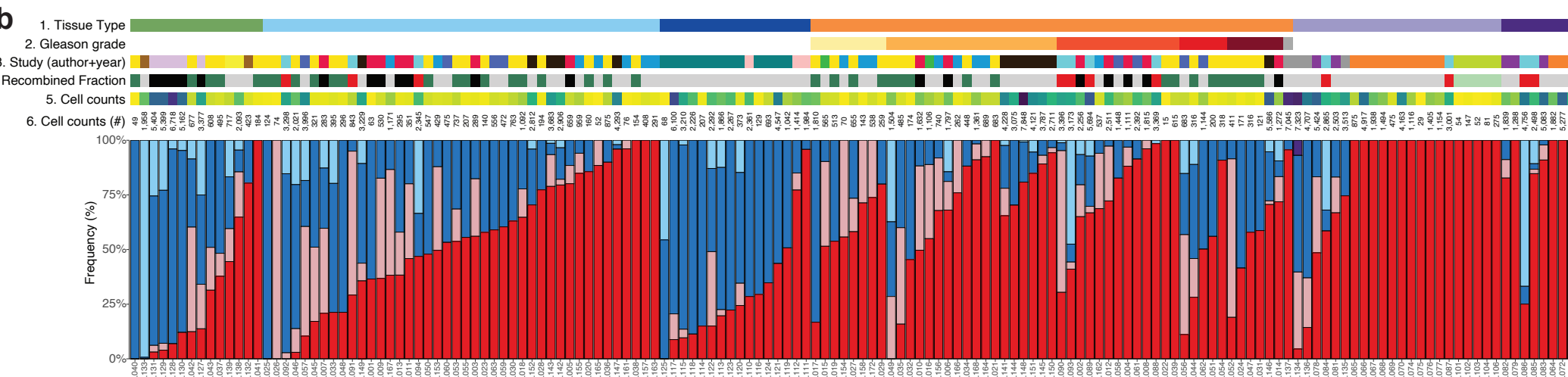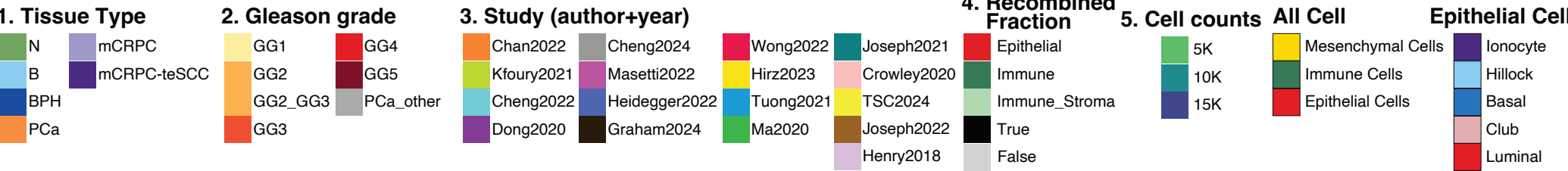

Cho et al. Suppl Figure 4

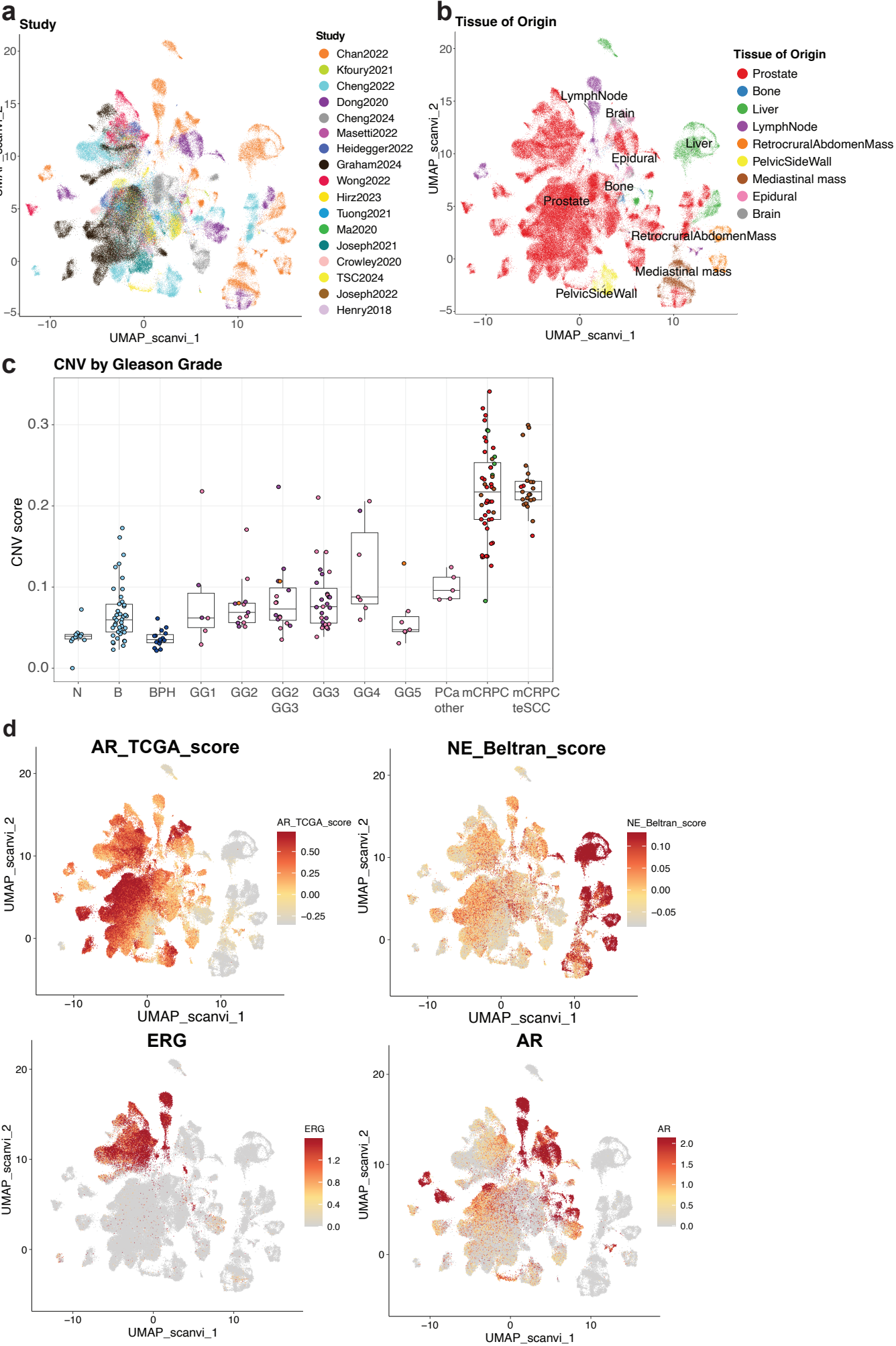

Supplement: Supplement 1 — Supplementary Figure 1. Quality control metrics and cell counts across processing stages. (a) Violin plots showing per-cell quality control metrics across 17 studies prior to filtering: UMI counts (nCount_RNA, log10 scale), detected gene counts (nFeature_RNA, log10 scale), and mitochondrial transcript fraction (Percent.mt). Red dashed lines indicate filtering thresholds applied per sample (UMIs ≥500, genes ≥250, MT% <20). Pink shading indicates the excluded region below (UMI, gene) or above (MT%) each threshold. (b) Boxplot of per-sample doublet rates (%) estimated by scDblFinder across all 17 studies. Each point represents one sample. (c) Bar plot showing the number of cells at each processing stage for each of the 17 studies. Bars represent cells at four stages: before QC filtering (orange), after QC filtering (yellow), after doublet removal (grey), and cells retained in the final dataset after annotation-based filtering (dark green). Total cells: 909,499 before QC; 847,090 after QC filtering; 767,524 after doublet removal; 754,195 in the final dataset. Sample-level counts are provided in Supplementary Table 2. Supplementary Figure 2. Study-level cell distribution across the integrated atlas. (a) UMAP embedding colored by study of origin, illustrating mixing of cells from all 17 datasets across the embedding. (b) Donut chart showing the proportion of cells contributed by each study. Hirz2023 contributes the largest fraction (26.1%), followed by Joseph2021 (13.9%) and Graham2024 (9.2%). Supplementary Figure 3. Sample-level cell type composition across all 163 samples. (a) Sample-level stacked bar plot showing the proportional composition of broad cell compartments (epithelial, immune, and mesenchymal) across all 163 samples. Metadata tracks above indicate tissue type, Gleason grade, study of origin, recombined fraction status, and total cell count per sample. Samples are ordered by tissue type and disease state. (b) Same layout as (a), showing proportional composit [file media-1.pdf]
